# Lowering the Carbon Footprint Through Telehealth Vasectomy Consults: A Retrospective Observational Study

**DOI:** 10.7759/cureus.70698

**Published:** 2024-10-02

**Authors:** Paul J Wurtz, Emma Harwood, Thomas E Schroeder, Christopher M Deibert

**Affiliations:** 1 Internal Medicine, University of Nebraska Medical Center, Omaha, USA; 2 Internal Medicine, Brooke Army Medical Center, Fort Sam Houston, USA; 3 Urology, University of Nebraska Medical Center, Omaha, USA; 4 Urology, Maine Medical Center, Portland, USA; 5 Urology, Duke University Medical Center, Durham, USA

**Keywords:** carbon footprint, climate change, telemedicine, vasectomy, virtual health

## Abstract

Objective: This study aimed to quantify the impact of shifting in-person pre-vasectomy consultations to telehealth visits on travel time and carbon footprint reduction.

Materials and methods: Utilizing retrospective chart analysis, we examined men who underwent vasectomy at our institution from November 15, 2020, to November 15, 2021. Using their home address, we estimated the distance and travel time with Google Maps direction services to our institution's outpatient urology clinic. We then quantified the number of miles of gas used by dividing that distance by 25 miles per gallon, based on the United States Environmental Protection Agency market average in 2020. We then estimated the pounds of carbon dioxide produced per gallon of gasoline using the United Energy Information Administration's estimate that each gallon of gasoline produces 18.74 pounds of carbon dioxide.

Results: After including 126 cases, the average patient saved 26 miles round trip between their home address and our institution's outpatient clinic. This averaged 43 minutes per round trip. Using our reference values, each trip saved an estimated 1.04 gallons of gasoline and 19.48 pounds of carbon being produced.

Conclusions: By transitioning pre-vasectomy consults from an in-person to a telehealth format, patients have added convenience in reduced travel in addition to a lower individual carbon footprint.

Impact statement: With an average of 500,000 vasectomies performed per year, transitioning from in-person to telehealth vasectomy pre-consults has the potential to save patients' travel time while reducing their carbon footprint.

## Introduction

As a major contributor to the global carbon footprint, the healthcare industry has been tasked with finding ways to cut back on greenhouse gas (GHG) emissions [1]. Estimates have indicated that healthcare facilities in the United States account for 8% of GHG emissions. In the United Kingdom, healthcare accounts for 30% of the GHG emissions from the public sector [2].

Carbon dioxide (CO2) is the dominant GHG emitted by healthcare-related activities and is responsible for 80-85% of the global warming potential (GWP) of the healthcare sector in the United States and United Kingdom. Other emitted GHGs include methane, nitrous oxide, chlorofluorocarbons, and anesthetic gases, which, together with CO2, can be converted into carbon dioxide equivalents (CO2e). The summation of these gases is a carbon footprint [3]. Worldwide, the carbon footprint of healthcare is estimated to account for 4.4% of the global total [4].

Of the many strategies to reduce the healthcare industry's carbon footprint, reducing transport emissions and costs has a large potential impact, as travel-related carbon emissions are estimated at 7% of the global healthcare footprint [4]. Patient transport vastly outnumbers the carbon footprint of staff and other transport-related healthcare emissions. A recent study from Greece found that up to 86% of transport-related healthcare carbon emissions are derived from individual patient transport to and from the healthcare facility [2]. 

One way to reduce GHG transport emissions is to transition in-person clinic visits to telehealth. This would largely reduce the number of outpatient visits to healthcare facilities, which according to the most recent data published by the Centers for Disease Control and Prevention (CDC) comprised 860 million outpatient visits in the United States in 2018 [5]. Telehealth is not feasible for all outpatient visits but can be implemented for those in which an in-person physical exam is not necessary. Due to the recent advances in technology, telehealth is becoming more commonplace in clinical practice. This shift was accelerated by the need for social distancing during the coronavirus disease 2019 (COVID-19) pandemic [6-9].

Telehealth provides patients with significant cost and time savings by reducing travel time and time away from work [10,11]. Telehealth also has the potential to reduce total appointment time since patients do not need to check in and rooming processes are nearly eliminated [8]. Additional studies have demonstrated that patient care is equal to that of in-person care and that patient-reported adverse events are not increased as a result of telehealth [11-13].

In urology, telemedicine has been successfully utilized for a variety of indications, such as pathology and/or imaging review, prostate cancer surveillance, urolithiasis, lower urinary tract symptoms, and vasectomy [6,7,10,12]. With an average of 500,000 vasectomies performed in the United States each year, transitioning pre-vasectomy consults to telehealth has the potential to reduce GHG emissions and lower the carbon footprint [14]. The quantitative effect of transport-related GHG emissions can be measured in the form of pounds of CO2 produced. The goal of our study was to quantify the carbon footprint of one of the most common procedures in urology and demonstrate how changing practice models from in-person to telehealth has the potential to decrease carbon emissions.

## Materials and methods

Data collection

We conducted a retrospective chart analysis to gather data from men who underwent vasectomy procedures at our institution in Omaha, Nebraska, between November 15, 2020, and November 15, 2021. In an effort to improve accessibility and convenience, telehealth consultations were preferentially offered to patients. From this group, we identified those who attended a virtual pre-vasectomy consultation via telehealth.

Distance and travel time estimation

The home addresses of these patients were used to estimate the distance and travel time to our institution's outpatient urology clinic. These estimations were carried out using Google Maps direction services. To ensure the accuracy and consistency of our data, the distance and travel time calculations were performed during non-peak hours, specifically during evenings and weekends, to minimize the influence of traffic-related variables.

Fuel consumption calculation

To quantify the environmental impact of travel, we calculated the number of miles traveled per round-trip transit. This distance was then divided by 25 miles to estimate the gallons of gasoline consumed per trip. The formula used is as follows: gallons of gasoline per trip=(total distance per round trip)/25.

This estimation is based on the United States Environmental Protection Agency (EPA)'s average fuel economy for cars manufactured in the year 2020, which is 24.9 miles per gallon [15].

Carbon footprint estimation

Next, we estimated the pounds of CO2 produced per round-trip transit. Using the previously calculated quantity of gallons of gasoline consumed per trip, we multiplied this figure by 19 to determine the pounds of CO2 produced. The formula applied is as follows: pounds of CO2 per trip=(gallons of gasoline per trip)×19.

This calculation is based on the United Energy Information Administration (EIA)'s estimate that each gallon of gasoline generates approximately 18.74 pounds of CO2 [16].

Financial savings analysis

Finally, we used $3.00 per gallon to calculate the total financial savings based on the number of gallons saved. This value was based on Nebraska's average retail gasoline prices in 2021 [17]. The formula used is as follows: financial savings (in US dollars)=(total gallons of gasoline)×3.00.

Data analysis

By utilizing Excel's computational capabilities, we were able to systematically analyze and compile the data to derive meaningful insights. We used it to perform the following calculations: total and average travel distance, total and average travel time total financial savings, total gasoline consumption, and total and average carbon footprint, as measured in pounds of CO2 produced. 

## Results

A total of 126 pre-vasectomy telehealth consultations were included in the analysis, as detailed in Table 1.

**Table 1:** Summary of telehealth pre-vasectomy consultations and associated savings

Total number of pre-vasectomy telehealth consultations	126
Miles saved	
Total	3328
Average per trip	26
Time saved (min)	
Total	5554
Average per trip	43
Cost savings (USD)	
Total	400
Fuel savings (gal)	
Total	133
Pounds of carbon dioxide saved	
Total	2494
Average per trip	19.48

Distance avoidance and time savings

On average, each patient avoided a round-trip distance of 26 miles by utilizing telehealth services. This resulted in an average time saving of 43 minutes per patient.

Financial benefits

The total cost savings achieved by avoiding travel amounted to $400, accounting for reduced expenditure on gasoline, vehicle maintenance, and other travel-related costs.

Environmental impact

The reduction in travel led to a total saving of 133 gallons of gasoline. This decrease in fuel consumption translated into a significant reduction in carbon emissions, with a total of 2494 pounds of CO2 emissions saved. On average, each avoided round trip resulted in a reduction of 19.48 pounds of CO2 emissions.

These results indicate that the adoption of telehealth for pre-vasectomy consultations contributed to substantial time and cost savings, as well as a reduction in environmental impact.

## Discussion

This study measured the reduction in carbon emission as a result of transitioning pre-vasectomy consults from in-person to telehealth. For 126 telehealth consults, a total of 2494 pounds of CO2 was saved. Data from several studies suggest that travel-related GHG emissions may be the major contributor to the healthcare industry's large carbon footprint. A recent British study from 2021 found that there was an estimated reduction of 6.07 tons of CO2 emissions based on the predicted carbon footprint of "car traveler" patients alone [5]. A recent study published in the International Journal of Environmental Research and Public Health found a total reduction of 29.384 tons of CO2 when looking at 12,322 telemedicine cases [18]. 

In our study, the total reduction in carbon footprint was significant, and given that this occurred on a small scale of 126 patients, it could be more impactful if implemented on a larger scale. If we were to extrapolate the average CO2 savings from our study and apply it to the 500,000 vasectomies performed yearly [14], this would equal nearly 50,000 tons of CO2. There have been other studies that have quantified the use of telehealth to reduce the carbon footprint, but there have not been any US studies that have addressed the implications of performing vasectomy consults exclusively via telehealth.

The GHG emissions saved by reducing travel may also be proportional to the distance a patient would need to travel to a healthcare facility. A recent systematic review published in the Royal College of Physicians found that the mean distance and emission savings per consultation were 334.912 km and 0.09555 tons CO2e for telemedicine services serving rural populations (compared with 167.79 km and 0.0377 tons CO2e for telemedicine services serving urban populations), supporting this idea [19]. Comparatively, our results demonstrated a more modest reduction in travel distance, which could be a result of a smaller sample size and less need for patients in rural communities to travel to cities for vasectomies. 

In addition to reducing the carbon footprint, transitioning to telehealth also offers the benefit of time and money saved secondary to reduced travel. We found that on average, patients saved 43 minutes by avoiding a round trip to the clinic from their home address. We found numbers ranging from 25 to 97 minutes in the literature that we reviewed [6,7,18,19]. A study also estimated a cost saving per patient ranging from $149 to $252 when considering both the cost of travel and time away from work [20].

One concern that may arise in transitioning to telehealth visits is the omission of a physical exam and the potential for a related adverse event. A 2020 study of virtual urology clinics found that none of their patients were recorded or identified as undergoing an adverse event as a direct result of their virtual consultation nor did any patients file formal complaints related to their virtual consultation [12]. As it relates to urology, of concern is the omission of a genitourinary exam. A recent study reported that there was no statistical difference in completion rates of in-office vasectomy when comparing the virtual consult cohort and the in-office consultation cohort [18]. In our practice, we counsel the patient on the possibility that the in-office vasectomy may not be completed if a physical exam at that time is concerning. Patients will need to be informed about the nature of telehealth consultations, including potential limitations such as the absence of physical examinations. So that the transition to telehealth does not compromise the quality of patient care, providers will need to frequently assess whether telehealth is appropriate for all patients and conditions and provide alternatives when necessary.

Regarding privacy, measures should be in place to ensure the Health Insurance Portability and Accountability Act (HIPAA) and privacy rights are not compromised and to ensure confidentiality during appointments. Additionally, we must consider the digital divide and ensure that all patients, including those from underserved or technologically disadvantaged populations, have access to telehealth services.

Limitations of this study include the method of calculating travel distance and time. We used Google Maps during "off-peak" time periods, which may have underestimated carbon emissions from sitting in longer traffic times. Conversely, we do not know that all patients traveled from their home address or elsewhere, nor do we know if alternative modes of transportation were used to commute (e.g., public transportation, biking, walking). We did not consider the car type or fuel type that may have been used, which would affect carbon emission calculations. 

We rely on retrospective chart analysis, which is generally reliable but may have inherent biases related to record accuracy and completeness. Additionally, the sample size of 126 cases is relatively small. Larger sample sizes would provide more robust data and help to generalize the findings. The study population consists of men who underwent vasectomy at a single institution. The sample is specific to those who were offered and chose telehealth consultations, which might introduce selection bias. Although a retrospective design is efficient for utilizing existing data, it can introduce biases related to historical data collection methods. Our sample is geographically limited to patients within the catchment area of the institution, which might affect the generalizability of the results to other regions with different population densities and healthcare access patterns. Finally, this study covers a specific period (November 15, 2020, to November 15, 2021), which includes the COVID-19 pandemic era, potentially affecting patient behavior and healthcare practices.

This study further adds to the literature that telehealth reduces carbon emissions. This encourages transitioning in-person clinic visits to virtual visits and strengthens the case that it is a viable and effective way to reduce the carbon footprint of the healthcare industry [21-23]. Future studies should focus on methods of obtaining more accurate calculations of healthcare-related GHG emissions. This will strengthen the evidence and encourage increased use of telemedicine when appropriate. 

## Conclusions

While this study provides valuable insights into the potential benefits of telehealth for pre-vasectomy consultations, considerations regarding ethics, reliability, and sampling techniques are crucial for the robustness and generalizability of the findings. Further studies with larger, more diverse samples and prospective designs will strengthen the evidence base. Switching pre-vasectomy consults from in-person to telehealth had a clear reduction in carbon emissions as measured by pounds of CO2 produced. The healthcare sector is responsible for significant carbon emissions, and this study should motivate urologists to consider transitioning vasectomy consults to telehealth visits. This has the advantage of both reducing carbon emissions and saving patients' time and money.
